# State of emergency medicine in Rwanda 2015: an innovative trainee and trainer model

**DOI:** 10.1186/s12245-015-0067-2

**Published:** 2015-06-16

**Authors:** Gabin Mbanjumucyo, Elizabeth DeVos, Simon Pulfrey, Henry M Epino

**Affiliations:** Department of Emergency Medicine, Masaka District Hospital, P.O. Box 3472, Kigali, Rwanda; University Teaching Hospital of Kigali, Kigali, Rwanda; Department of Emergency Medicine, University of Florida College of Medicine-Jacksonville, 655 West 8th Street, Jacksonville, FL USA; Department of Emergency Medicine, University of British Columbia, St. Paul’s Hospital, 1081 Burrard Street, Vancouver, BC Canada; Department of Emergency Medicine, Massachusetts General Hospital, Zero Emerson Place, Boston, MA USA

**Keywords:** Rwanda, Emergency medicine, Postgraduate training, Global health, International emergency medicine, Trainer, Trainee, Emergency department

## Abstract

The 1994 Rwandan war and genocide left more than 1 million people dead; millions displaced; and the country’s economic, social, and health infrastructure destroyed. Despite remaining one of the poorest countries in the world, Rwanda has made remarkable gains in health, social, and economic development over the last 20 years, but modern emergency care has been slow to progress. Rwanda has recently established the Human Resources for Health program to rapidly build capacity in multiple sectors of its healthcare delivery system, including emergency medicine. This project involves multiple medical and surgical residencies, nursing programs, allied health professional trainings, and hospital administrative support. A real strength of the program is that trainers work with international faculty at Rwanda’s referral hospital, but also as emergency medicine specialty trainers when returning to their respective district hospitals. Rwanda’s first emergency medicine trainees are playing a unique and important role in the implementation of emergency care systems and education in the country’s district hospitals. While there has been early vital progress in building emergency medicine’s foundations in Rwanda, there remains much work to be done. This will be accomplished with careful planning and strong commitment from the country’s healthcare and emergency medicine leaders.

## Background

Rwanda is one of Africa’s smallest and most densely populated countries (368 inhabitants per square kilometer). Over half of its 10.6 million people are under 18 years of age [1]. The 1994 war and genocide left more than 1 million people dead, millions displaced, and the country’s economic and social infrastructure destroyed. Despite remaining one of the poorest countries in the world (GDP ranking 166/187), Rwanda has made remarkable gains in health, social, and economic development over the last 20 years [2].

## Health care

Over 40 % of the population lives in extreme poverty and suffers significant morbidity and mortality from communicable diseases [3]. While HIV/AIDS, acute respiratory infections, diarrheal diseases, and tuberculosis account for most Rwandan health facility patient visits, noncommunicable disease (NCD) burden is increasing [4]. Particularly, road traffic injuries account for many of the disability-adjusted life years (DALYs) lost in Rwanda [5]. Natural disasters and ongoing regional conflicts also disrupt some care delivery.

A combination of state funds, health insurance programs, and direct fee for services finance the Rwandan healthcare system. Foreign aid funds 53 %. Approximately 80 % of Rwandans have some form of health insurance to offset acute care costs [6]. The Mutuelle de la Sante is the largest and most basic social health insurance program. Members pay annual premiums of approximately USD $6 per family member, and 10 % co-pay for each health system encounter.

Rwanda’s multitiered healthcare system utilizes over 430 health centers to provide nurse-supervised primary care. These clinics also oversee multiple health posts providing immunizations and antenatal care. Through these clinics, community health workers (CHWs) monitor village level health and refer sick individuals to the nearest health facility. These health centers are supported by 44 district hospitals that provide inpatient and outpatient care. Four national referral hospitals provide specialty care [7].

## Accessing emergency care in Rwanda

Although some emergency cases present first to health centers where emergency equipment and skills are very limited, most emergency patients are expedited to district hospital care. General practitioners, typically recent medical school graduates with no formal postgraduate level training, usually staff these wards [8]. Cases requiring complex care are transferred to a referral hospital. Acute medical and trauma cases may present directly to a district hospital or a national referral hospital by private vehicle or ambulance.

## Discussion

Rwanda has established the Human Resources for Health (HRH) program to rapidly build capacity in multiple sectors of its healthcare delivery system [9]. This exciting project involves multiple foreign medical and surgical residencies, nursing programs, allied health professional trainings, and hospital administration support. Partnered with sidHARTe (Systems Improvement at District Hospitals and Regional Training of Emergency Care), HRH is providing an emergency medicine (EM) residency-training program. This two-tiered system addresses an immediate need for trained EM practitioners and specialists. The first tier consists of a 2-year, part-time postgraduate diploma (PGD) course in emergency and critical care medicine to create capacity for establishing emergency medicine at the country’s 44 district hospitals. The second tier will recruit graduates from the PGD course to continue training for another 3 years to earn a master of medicine in emergency medicine. The first nine PGD participants began their EM training in September 2013. Over 2 years, PGD participants will spend four 3-month blocks (total 12 months) at the public referral hospital in Kigali. After each block, candidates return to their home hospitals for 3 months where they are encouraged to implement emergency system and care improvements.

A real strength of the PGD program is having trainers both work with international faculty at the referral hospital, but also as EM specialty trainers when returning to blocks of work in their respective district hospitals. Unlike trainees in North American and European EM systems, PGD participants are leaders in their district hospitals from as early as the fourth month of their training. They work both to provide direct patient care and also to implement new emergency care policies. District hospitals where current PGD participants work are slowly developing properly equipped and functioning emergency departments. PGD candidates also work diligently to educate district hospital staff and administrators who are often not yet aware of the benefits of establishing effective emergency systems.

With sidHARTe support, candidates have introduced triage and resuscitation skills to their district hospital nursing and physician colleagues. In many cases, they have also changed local protocols and systems to better deliver emergency care. The opportunity to participate as candidates and trainers for an important new specialty is both exciting and meaningful.

Although there is not yet formal data, many PGD participants feel that some of the morbidity and mortality at the district hospital level have decreased as a direct result of their training and implementing emergency medicine best practices. These positive outcomes and system changes are motivating to both PGD participants and their district level supervisors. For instance, one district hospital director responded to these EM successes by dedicating discretionary funds to disseminate emergency triage training to the health centers within his hospital’s catchment area.

Emergency medicine in Rwanda faces the challenge of needing to prove its worth in terms that matter to the country’s public health policy makers. To do this, new emergency systems will need to collect and analyze data to prove direct impact of care on outcomes. Otherwise, further difficulties for EM’s development in Rwanda at the hospital level include inpatient bed access blockage, maintenance of adequate emergency equipment and supplies, and the reluctance of some hospital services to accept a role for emergency medicine in their hospitals [10]. Other immediate challenges for the continued growth of EM care in Rwanda include establishing specific EM nursing and developing adequately trained and resourced emergency medical first response systems [8].

## Conclusion

Emergency medicine is still a nascent field in Rwanda. Rwanda’s first EM trainees are already playing a unique and important role in the implementation of emergency care systems and education in the country’s district hospitals. While there has been exciting and vital progress in building its foundations, there remains much work to be done. This will be accomplished with careful planning and strong commitment from the country’s EM and healthcare leaders.

## Abbreviations

CHWs: Community health workers
DALYs: Disability-adjusted life years
EM: Emergency medicine
GDP: Gross domestic product
HRH: Human resources for health
NCD: Noncommunicable disease
PGD: Postgraduate diploma
sidHARTe: Systems improvement at district hospitals and regional training of emergency care
USD: United States dollar
